# Changing clinical practice: management of paediatric community-acquired pneumonia

**DOI:** 10.1111/jep.12091

**Published:** 2013-10-07

**Authors:** Mohamed A Elemraid, Stephen P Rushton, Matthew F Thomas, David A Spencer, Katherine M Eastham, Andrew R Gennery, Julia E Clark, North East of England Paediatric Respiratory Infection Study Group

**Affiliations:** 1 Paediatric Registrar and Research Fellow, Department of Paediatric Infectious Disease and Immunology, Newcastle upon Tyne Hospitals NHS TrustNewcastle, UK; 2 Consultants in Paediatric Infectious Disease and Immunology, Department of Paediatric Infectious Disease and Immunology, Newcastle upon Tyne Hospitals NHS TrustNewcastle, UK; 3 Paediatric Registrar and Research Fellow, Institute of Cellular Medicine, Newcastle UniversityNewcastle upon Tyne, UK; 4 Consultants in Paediatric Infectious Disease and Immunology, Institute of Cellular Medicine, Newcastle UniversityNewcastle upon Tyne, UK; 5 Professor of Biological Modelling, School of Biology, Newcastle UniversityNewcastle upon Tyne, UK; 6 Paediatric Registrar and Research Fellow, School of Biology, Newcastle UniversityNewcastle upon Tyne, UK; 7 Paediatric Registrar and Research Fellow, Department of Respiratory Paediatrics, Newcastle upon Tyne Hospitals NHS TrustNewcastle upon Tyne, UK; 8 Consultant Respiratory Paediatrician, Department of Respiratory Paediatrics, Newcastle upon Tyne Hospitals NHS TrustNewcastle upon Tyne, UK; 9 Department of Paediatrics, Sunderland Royal HospitalSunderland, UK

**Keywords:** antibiotics stewardship, antibiotics, children, investigations, management guidelines, pneumonia

## Abstract

**Rationale and aim:**

To compare clinical features and management of paediatric community-acquired pneumonia (PCAP) following the publication of UK pneumonia guidelines in 2002 with data from a similar survey at the same hospitals in 2001–2002 (pre-guidelines).

**Methods:**

A prospective survey of 11 hospitals in Northern England was undertaken during 2008–2009. Clinical and laboratory data were recorded on children aged ≤16 years who presented with clinical and radiological features of pneumonia.

**Results:**

542 children were included. There was a reduction in all investigations performed (*P* < 0.001) except C-reactive protein (*P* = 0.448) between surveys. These included full blood count (76% to 61%); blood culture (70% to 53%) and testing of respiratory secretions for viruses (24% to 12%) and bacteria (18% to 8%). Compared to pre-guidelines, there was a reduction in the use of intravenous antibiotics as a proportion of the total prescribed from 47% to 36% (*P* < 0.001) and a change in the route of antibiotic administration with increasing preference for oral alone (16% pre-compared to 50% post-guidelines, *P* < 0.001).

**Conclusion:**

Apart from the acute phase reactants that should not be measured routinely, these changes are in line with the guideline recommendations. Improvements in antibiotic use are possible and have implications for future antimicrobial stewardship programmes. Further work using cost-effectiveness analysis may also demonstrate a financial benefit to health services from adoption of guidelines.

## Introduction

Paediatric community-acquired pneumonia (PCAP) is a frequent cause of admission to hospital 1,2. Clinical features of pneumonia are often non-specific in young children 3,4. Management decisions are generally based on a combination of clinical signs, symptoms and radiological changes 3,5. National UK clinical guidelines for management of PCAP were published in 2002 6 and updated in 2011 1 by the British Thoracic Society (BTS). They synthesized evidence and expert opinion to produce best practice national standards, which included statements on investigations and antibiotics use (Table 1) 6.

**Table 1:** Selected standards from 2002 British Thoracic Society management guidelines of PCAP 6

Blood cultures should be performed in all children suspected of having bacterial pneumonia. Nasopharyngeal aspirates from all children under the age of 18 months should be sent for viral antigen detection with or without viral culture. Acute phase reactants should not be measured routinely. Amoxicillin is first choice for oral antibiotic therapy in children aged <5 years and macrolide antibiotics may be used as first line empirical treatment in children aged ≥5 years. Antibiotics administered orally are safe and effective for children presenting with CAP. Intravenous antibiotics should be used in the treatment of pneumonia in children when the child is unable to absorb oral antibiotics (for example, because of vomiting) or presents with severe signs and symptoms. Appropriate intravenous antibiotics for severe pneumonia include Co-amoxiclav, Cefuroxime, and Cefotaxime. If clinical or microbiological data suggest that *Streptococcus pneumoniae* is the causative organism, Amoxicillin, Ampicillin or Penicillin alone may be used.

Antibiotic stewardship programmes and management guidelines have been shown to improve the selection of appropriate investigations and antibiotics for management of infections in children 7–11. These measures allow better use of health resources and reduction of antibiotic drug resistance which are becoming global challenges 12–17.

In the UK, the 7-valent pneumococcal conjugate vaccine (PCV7) was introduced routinely from September 2006. It was associated with a reduction in the incidence of PCAP and rate of hospitalization 18. We therefore aimed to explore changes in the management of children with pneumonia seen in hospital in the context of the national guidelines. Presentation and management outcomes of pneumonia in children in the present survey were compared to those previously described in a similar survey conducted in the same region in 2001–2002 3, prior to the publication of the BTS management recommendations for PCAP in 2002 6. Such findings are important for doctors involved in the management of this infection and for experts updating these guidelines.

## Methods

### Participants

A prospective survey of children aged ≤16 years who presented with clinical and radiological features of pneumonia at 11 hospitals (sites) was conducted in Northern England (excluding Cumbria) from August 2008 to July 2009 ‘post-guidelines’. Pneumonia was defined as any child with signs or symptoms suggestive of lower respiratory tract infection including any of fever, tachypnoea (defined age-specific respiratory rates), dyspnoea, cough, respiratory distress, chest wall retractions and auscultatory findings such as crackles, bronchial breathing or reduced breath sounds together with chest radiographic findings consistent with pneumonia as determined initially by the local paediatrician. Exclusions included being resident outside the geographical study area; clinical bronchiolitis; hospital admission within 3 weeks of pneumonia admission; or normal chest radiograph. Disease severity was classified according to the BTS criteria 6. Chest radiographic changes from the local radiologists' reports were classified into patchy, lobar or perihilar consolidations according to the World Health Organization criteria 19. ‘Non-end-point changes’ such as increased bronchovascular markings, peribronchial thickening, bronchial wall thickening or peribronchial cuffing were grouped together in an additional category ‘other infiltrates/abnormalities’. Exclusions included being resident outside the geographical study area; clinical bronchiolitis; hospital admission within 3 weeks of pneumonia admission; or normal chest radiograph. This cohort was included in a region-wide survey investigating the impact of PCV7 on the disease incidence and rates of hospitalization of pneumonia confirmed on chest radiograph 20.

Results were compared with those from an identically performed survey, using the same recruitment methods and diagnostic criteria in 2001–2002 ‘pre-guidelines’ 3. Hospital reconfigurations reduced the number of units admitting children from 13 to 11 during the pre- and post-guidelines surveys, respectively. The catchment population and referral pathways from primary care or accident and emergency departments to paediatric services remained the same. Ethical approval was obtained from the Newcastle and North Tyneside Research Ethics Committee. Caldicott approvals regarding data protection and information governance were granted from all collaborating sites.

### Data collection and case ascertainment

The study team carried out multiple visits to collaborating sites in order to explain and ascertain the adherence to the enrolment criteria through a locally identified junior and consultant paediatrician at each site. These visits also included supporting the Caldicott procedures, cross-checking and safe handling of data and presenting collected results. A family doctor/general practitioner or medical staff at the accident and emergency departments saw children before referral for further assessment by the paediatric team. Children were managed entirely by their local paediatric team. Data were recorded on standard form and validated by reviewing ward admission diaries for children admitted with respiratory symptoms (eight sites), or by obtaining hospital coding data on pneumonia where admissions are carried out electronically (three sites). Hard copy case notes and electronic records were reviewed to ascertain the data and resolve any missing or inconsistent data. Duplicates or those who did not fulfil the enrolment criteria were removed after the completion of validation process.

### Statistical analysis

Descriptive analysis was performed using Epi Info™ 7 (Centers for Disease Control and Prevention, Atlanta, GA, USA). Fisher's exact test with odds ratios (ORs) and 95% confidence intervals (CIs) was used to compare categorical variables between groups and with those from the pre-guidelines survey 3. Continuous variables are presented as mean ±standard deviation (SD). Incidence rates per 10 000 children were established for overall rates of pneumonia, hospitalization, disease severity and radiological findings and compared between the pre- and post-guidelines surveys using the population estimates for the North East Strategic Health Authority area from the UK Office of National Statistics for 2009 21. There were 458 500 children aged under 16 years. CIs of incidence rates were calculated assuming a Poisson distribution and using the EpiTools package in R statistical software version 2.15.1 (The R Foundation for Statistical Computing, Vienna, Austria). SPR provided guidance and supervision on data analysis.

## Results

A total of 542 were eligible for inclusion (58% males; 74% < 5 years old). Overall, 84% children were admitted (89% pre-guidelines). None of those who were discharged home after initial assessment returned to hospital within 3 weeks with clinical features suggestive of lower respiratory tract infection. Ten children required admission to the intensive care for assisted ventilation; eight were aged <5 years. An underlying co-morbidity was present in 15% and asthma in 7%. No children died during either survey periods. The epidemiological outcomes for this cohort are described in a separate publication 20. The overall reduction in annual incidence of pneumonia per 10 000 children between 2001 and 2009 was 17.7% (95% CI 8 to 26); a similar reduction was observed in annual hospitalization rate 18.5% (95% CI 8 to 28) 20. Table 2 compares data between the pre- and post-guidelines surveys on clinical and radiological features.

**Table 2:** Comparison of rates of pneumonia, hospitalization, disease severity and radiological findings between the two surveys per 10 000 children

	2001 survey (*n* = 711)		2009 survey (*n* = 542)		Change in IR*
Variables	*n* (%)	IR (95% CI)	*n* (%)	IR (95% CI)	% (95% CI)
Pneumonia	711 (100)	14.4 (13.4 to 15.4)	542 (100)	11.8 (10.9 to 12.9)	17.7 (8 to 26)
Hospitalization	636 (89.5)	12.2 (11.3 to 13.2)	455 (84.0)	9.9 (9.0 to 10.9)	18.5 (8 to 28)
Pre-admission antibiotics	214 (30.0)	4.1 (3.6 to 4.7)	119 (22.0)	2.6 (2.2 to 3.1)	36.7 (20 to 49)
Disease severity					
Mild/moderate	293 (41.2)	5.6 (5.0 to 6.3)	259 (47.8)	5.7 (5.0 to 6.4)	−0.7 (–19 to 15)
Severe	418 (58.8)	8.0 (7.3 to 8.8)	283 (52.2)	6.2 (5.5 to 6.9)	22.9 (10 to 34)
Chest radiographic findings					
Lobar	145 (20.4)	2.8 (2.3 to 3.3)	162 (29.9)	3.5 (3.0 to 4.1)	–27.2 (–60 to −1)
Patchy	436 (61.3)	8.4 (7.6 to 9.2)	296 (54.6)	6.5 (5.7 to 7.2)	22.7 (20 to 33)
Perihilar	130 (18.3)	2.5 (2.1 to 2.9)	67 (12.4)	1.5 (1.1 to 1.9)	41.3 (20 to 57)
Other infiltrates	–	–	17 (3.1)	0.4 (0.2 to 0.6)	–
Pleural effusion	65 (9.0)	1.2 (0.9 to 1.6)	52 (9.6)	1.1 (0.9 to 1.5)	8.9 (–33 to 38)

* Negative numbers denote an estimate of an increase in incidence.

CI, confidence interval; IR, incidence rate.

### Presentation

Among those with pleural effusion, reported lobar changes were present in 77% post-guidelines compared to 42% pre-guidelines (OR = 0.2; 95% CI 0.09 to 0.48; *P* = 0.0002). Pyrexia (triage temperature >38°C) was recorded in 47.4% (333 out of the 702) pre-guidelines and 50% (266 out of the 531) post-guidelines (*P* = 0.358). Rates of admission oxygen saturation <93% were similar between the pre- (31%, 213 out of the 689) and post-guidelines surveys (27.4%, 145 out of the 529) (*P* = 0.204). Hospitalization was associated with disease severity (*P* < 0.001), but not with pyrexia or chest radiographic changes.

### Investigations

There was an association between the collection of blood samples for investigation(s) and use of intravenous (IV) antibiotics pre-guidelines (*P* < 0.001), but not post-guideline. There was a reduction in the number of investigations performed (*P* < 0.001) except C-reactive protein (CRP) (*P* = 0.448) between pre- and post-guidelines. Full blood count (FBC) decreased from 76% to 61%; blood culture from 70% to 53%; testing respiratory secretions for viruses from 24% to 12% and bacteria from 18% to 8%. The yield of blood culture was the same in both surveys (4% and 4.9%). Collection of blood culture post-guidelines was not correlated to disease severity (*P* = 0.085). Post-guidelines, viral polymerase chain reaction assays (immunofluorescence test in pre-guidelines) were performed on respiratory secretions from 66 children with 26 (39%) positive. Obtaining a viral respiratory screen was age-dependent and more frequently performed in those aged <2 (22%) than ≥2 years, but less often when compared with pre-guidelines (34%) (OR = 0.5; 95% CI 0.33 to 0.75; *P* = 0.001).

CRP was obtained in 322 (59%); of which, 27% were >100 mg L^−1^; 9% of infants, 58% of under 5 years old and 42% in the above five. Pleural effusion was associated with higher CRP greater than 100 mg L^−1^ (*P* < 0.001). Lobar and patchy changes were associated with a CRP more than 150 mg L^−1^ (*P* < 0.05). Mean values of CRP, total white cell count and neutrophils were higher with lobar changes (*P* < 0.001).

### Management

Between the pre- and post-guidelines, IV antibiotics as a proportion of the total prescribed antibiotics decreased from 47% (501 out of the 1065) to 36% (318 out of the 891) (OR = 1.6; 95% CI 1.33 to 1.93; *P* < 0.001), and oral antibiotics alone increased from 16% to 50% (OR = 4.4; 95% CI 3.37 to 5.71; *P* < 0.001). There was also a reduction in the use of IV route only from 8% to 5% (OR = 1.8; 95% CI 1.08 to 2.86; *P* = 0.025) and the use of both oral and IV routes (*P* < 0.001) between the pre- and post-guidelines, respectively. Post-guidelines, Amoxicillin prescription both orally and intravenously increased (*P* < 0.001) with a decrease in IV cephalosporins (Cefuroxime and Cefotaxime) (*P* < 0.001) and total oral macrolides (Erythromycin, Azithromycin and Clarithromycin) (*P* < 0.001). However, the individual use of Azithromycin or Clarithromycin remained the same, while decreased for Erythromycin (*P* < 0.001).

Pre-guidelines, initial IV antibiotics were associated with severe disease, lobar changes, pleural effusion or pyrexia (*P* < 0.05), but not with oxygen saturation <93%. These associations were replicated in post-guidelines with the initial use of IV antibiotics being associated with severe disease (*P* = 0.0003), lobar changes (*P* = 0.018) or pleural effusion (*P* = 0.041), but not with oxygen saturation <93% or pyrexia. Comparing post- with pre-guidelines; IV antibiotics were more likely to be given to those with lobar changes (35% versus 25%) (OR = 0.6; 95% CI 0.45 to 0.85; *P* = 0.004), but less likely to be given to children presenting with low oxygen saturations (25% versus 34%) (OR = 0.6; 95% CI 0.45 to 0.89; *P* = 0.009). Mean (±SD) duration of hospitalization decreased from the pre- to post-guidelines surveys (4.7 ± 7.16 versus 3.2 ± 3.02 days, *P* < 0.001). Those with severe disease, lobar changes or pleural effusion had a longer stay (*P* < 0.001). All children irrespective of their age group who received any IV antibiotics (alone or in combination with oral) had a longer average duration of hospitalization than those who had only oral (4.1 ± 3.4 versus 2.0 ± 1.9 days, *P* < 0.001).

## Discussion

This survey provides invaluable evaluation of the presentation and management of PCAP seen in hospital over a year period focusing on the investigations performed and antibiotic selection. Clinical management of children with pneumonia has changed significantly between 2002 and 2008. There have been a reduced number of investigations performed, a change in the type of antibiotics, a decrease in IV and a concomitant increase in oral antibiotics, and a reduction in hospitalization rates and duration of stay. Reasons for these changes are likely to be multifactorial such as the publication of the BTS management guidelines 6, an expanding literature on oral/IV antibiotic use 22–25, and the routine introduction of PCV7 in the UK in 2006. Findings from the present survey are an important step in supporting the improvement in adherence to the latest 2011 management guidelines 1.

Drivers of change are complex; some are likely to be literature driven and others probably reflect the complex relationships around perceived benefits and risks of IV cannulation, venepuncture and differing usefulness of investigations. Adherence to practice guidelines can be improved by regional studies coupled with continuing education, feedback of audit data and networking of junior and senior staff 26,27. Our regional respiratory diseases research group and respiratory network consists of staff from different disciplines, general and respiratory paediatricians from the local hospitals around the region with regular meetings, feedback and active research activities. Beside these activities, participation in two surveys on the management of PCAP helped raise awareness of management guidelines in all centres. In contrast, a recent national BTS audit of a 3-year data from 2009/2010 to 2011/2012 on the management of PCAP in the UK showed an overuse of investigations to diagnose pneumonia and under-prescription of oral antibiotics particularly Amoxicillin 28.

It is interesting that fewer blood tests in terms of FBC and blood cultures were taken, but just as many CRP samples were ordered. The BTS guidelines including the recently updated version in 2011 1 made no specific recommendations around FBC and discouraged use of acute phase reactants. Blood cultures were specifically encouraged for those suspected of having a bacterial pneumonia in 2002 6 but changed in 2011 1 to be performed only in those who presented with severe pneumonia. In the present survey, the correlation between high CRP of >100 mg L^−1^ with pleural effusion demonstrates the usefulness of CRP in differentiating between uncomplicated and complicated pneumonia of bacterial aetiology 29. The reduction in collecting blood cultures perhaps reflects the feeling that bacterial pneumonia is less likely given the introduction of PCV7, which in the same population was associated with decreased disease incidence and rate of hospitalization 20. Although clinicians were not asked directly, the shift towards less testing of respiratory secretions for either viruses or bacteria could reflect the feeling that the results would not affect the decision on antibiotic use.

More positive changes are seen with antibiotic usage, encouraging for developing antimicrobial stewardship programmes. These included a significant reduction in the use of antibiotics prior to admission. This is in line with the observed substantial decline since 1990s in the prescription of antibiotics in primary care for lower respiratory tract infection in children 30. This fall in antibiotic prescriptions predate the published BTS management guidelines of pneumonia in 2002 6. They reflect a continued fall in the use of antibiotics despite a marginal increase in antibiotic prescription during the period between 2003 and 2006, primarily for non-specific upper respiratory tract infections, for which national guidance aimed at primary care was introduced in 2008 30,31. IV antibiotics were used far less frequently than oral, with a substantial increase in the use of Amoxicillin overall and orally, at the expense of IV Cefuroxime and oral cephalosporins, which decreased from one-fifth to 2%. In contrast, oral macrolides remain frequently prescribed particularly to those aged under five, similar to previous data 3, although not recommended as first line treatment 6. Evidence for the safety and efficacy of oral antibiotics even in severe pneumonia in children accumulated over the 6 years period between surveys, including a Cochrane review in 2006 23 and the PIVOT trial in 2007 24. Recent pooled review data showed that even severe pneumonia in young children can be managed safely with oral antibiotics in primary care settings 25.

The selection of initial antibiotic route was influenced by disease severity and lobar changes, possibly reflecting that these criteria were considered markers of bacterial infection. The fact that lobar changes were associated with high mean value of inflammatory markers may support this. Other factors that could have influenced the decision to give IV antibiotics, such as the level of training of admitting medical staff or the knowledge of the published guidelines, could not be ascertained with the data collected. Substantial variation in the prescription of antibiotics for children has been described, including both the proportion of children exposed to antibiotics (38%–72%) and the duration of treatment 32. Also, considerable variability of antibiotic selection for the management of PCAP has been observed among paediatric infectious disease consultants 33. This variability in antibiotic use highlights the need to implement and monitor effective antibiotic stewardship policies across and within hospitals to reduce the over or under-use of them, thus reducing the risks of development of antibiotic-resistant bacteria and treatment failures 34.

## Conclusions

There has been a positive change in the management practices of PCAP reflected by reduced number of overall investigations performed and an increased preference for oral antibiotic use. This was associated with decreased admission rates and shorter length of hospital stay without increased complications. Adherence to practice guidelines can be improved by regional projects coupled with continuing education and feedback and networks of staff at all grades. A cost-effectiveness analysis focusing on the impact of reduced hospitalization, IV antibiotic use and pre-admission antibiotics would provide useful economic information on social cost.

## Authorship

JEC developed the survey concept and with KME and MAE were responsible for the survey logistics and facilitation of data collection. MAE managed and validated the data. MAE performed statistical analysis under guidance and supervision by epidemiologist SPR. All authors were involved in the interpretation of the results and writing of this article.

## Funding and conflict of interest

This survey was supported by a grant from Pfizer Vaccines UK (No: 0887X1-4479). The sponsor had no role in the survey design and data analysis or interpretation. JEC and DAS received unconditional research support from the Pfizer.
